# Whole ureter replacement with Yang–Monti principle: successful treatment of challenging conditions

**DOI:** 10.1186/s12894-022-01150-0

**Published:** 2022-12-08

**Authors:** Chyau-Wen Lin, Jen-Chieh Chen, William J. Huang, Tzu-Ping Lin

**Affiliations:** 1 Department of Urology, Taipei Veterans General Hospital, No. 201, Sec. 2, Shipai Rd., Beitou District, Taipei, 11217 Taiwan, R.O.C.; 2 Department of Urology, School of Medicine, National Yang Ming Chiao Tung University, No.155, Sec 2, Linong St., Taipei, 112304 Taiwan, R.O.C.

**Keywords:** Ileum, Split renal function, Ureteral reconstruction, Whole ureter

## Abstract

**Background:**

No clear consensus has been reached on the reconstruction of long-segment or total ureter discontinuation. Here we present our experience using the Yang–Monti technique in total ureter reconstruction.

**Methods:**

This study was a single-center retrospective study of patients who underwent Yang–Monti ileal whole ureter reconstruction (from the ureteropelvic junction[UPJ] to the ureterovesical junction). Data were collected on patients’ baseline characteristics, stricture etiology, the time interval between insult and surgical repair, pre/postoperative serum creatinine, estimated glomerular filtration rate (eGFR), split renal function, complications during admission and follow-ups, and the indwelling durations of JJ tubes and nephrostomy tubes, if presented.

**Results:**

Seven patients underwent Yang–Monti ileal ureter reconstruction in 2010–2020 at our hospital. One of the patients underwent single-session bilateral ureter repair. Radiation therapy-related fibrosis and degloving injury were the most common etiologies for ureter injury. The median interval between ureter insult and operation was 8 months. The median follow-up was 36.7 months. The average operation time was 11.4 h, and the average blood loss was 273 ml. Postoperatively, no significant differences were found in serum creatinine, eGFR, or split renal function. As for postoperative complications, two patients experienced ileus and were treated conservatively. One patient had UPJ stenosis, which resolved after re-anastomosis surgery 11 months later. Metabolic acidosis or electrolyte imbalance was not reported.

**Conclusion:**

We found that ileal replacement of total ureteral loss using the Yang–Monti principle is effective and durable. This is the largest cohort study conducted with more than 2 years of follow-up.

## Background

Ureter injuries are one of the common yet troubling issues in urology. Iatrogenic, radiation, and idiopathic retroperitoneal fibrosis are among the common etiologies that result in ureteral obstruction [1].

Repair or reconstruction with native urinary system tissue is preferred if feasible. Several strategies can be used for short-segment ureter injury or disruption, depending on location. Ureteroureterostomy, psoas hitch, and Boari flap are common interventions used to overcome ureter defects in the middle or lower third of the ureter.

However, long-segment, and even total ureter discontinuation, or high-level ureteral injuries are more problematic, and no clear consensus has been reached on reconstruction. Auto-transplantation, transureteroureterostomy, or intestinal replacement has been described in case reports or case series [2, 3]. Nevertheless, long-term follow-up with convincing data, such as fraction renal function, is sparse. Yang–Monti ileal ureter is a reconfigured ileal tube, which has been employed in long segment of ureter reconstruction. Here, we present our experience of Yang–Monti ileal ureter replacement for ureter reconstruction from the ureteropelvic junction (UPJ) to the ureterovesical junction (UVJ).

## Methods

### Participants

We identified all patients who underwent the Yang–Monti procedure from 2010 to 2020 at the Taipei Veterans General Hospital using the electronic medical records system. The following data were collected: patients’ baseline characteristics, stricture etiology, the time interval between insult and surgical repair, the intestinal length used in ureter substitutes, preoperative blood tests and images, postoperative serum creatinine levels, complications during admission and follow-ups, and the indwelling durations of JJ tubes and nephrostomy tubes if presented.

The common manifestation of these patients was flank pain on the affective side. Elevated serum creatinine was observed in four patients, but not all of them.

After the diagnosis of ureter stricture, endoscopic examination and intervention was first considered. Nearly all our patients had received diagnostic ureteroscopy or JJ tube insertion prior to the surgery. In the meantime, pre-operative assessment would be done, including patient’s performance status, presence of intractable urinary tract infection and underlying malignancy status if presented would be performed. We also discussed with the patients about other treatment choice. The operation would be performed if feasible.

All patients underwent the baseline workup, including serum creatinine and split renal function. The estimated glomerular filtration rate (eGFR) was calculated using the Modification of Diet in Renal Disease formula. Differential renal function was obtained by pre- and postoperative Tc-99 m MAG3 renal imaging. Follow-ups were conducted in the outpatient setting with serum creatinine concentration check-ups and imaging follow-ups if necessary. Complications were identified and classified according to the Clavien–Dindo Classification.

The study was approved by the Ethics Committee of Taipei Veterans General Hospital (IRB number: 2021–09-017AC.)

### Preoperative preparation

Due to the complexity and length of stricture in our patients, nephrostomy tubes were inserted in every patient prior to the reconstruction to maximally preserved the already compromised ipsilateral kidney function. Those tubes are left in place after the ileal tubes were matured and served as a tract for antegrade pyelography after removal of JJ stents.

The patients were given a low-residue diet 2 days before the operation and underwent bowel preparation 1 day before the operation.

### Surgical procedure

All of the surgeries were conducted under general anesthesia, and arterial line and central venous catheters were inserted for perioperative monitoring. We made a midline incision and entered the retroperitoneal space by incising the line of Toldt, followed by medial mobilization of the colon. We identified the upper limit of ureteral obstruction and dissected the renal pelvis above the obstruction. Next, we developed the prevesical space and exposed the dome of the bladder. We did not dissect the injured native ureter. We then harvested the ileal tube at least from 15 cm proximal to the ileocecal valve. We generally utilize five to six 2.5-cm long bowel segments to reform the ileal tube according to the Yang–Monti principles as described previously [4]. In brief, the bowel with the supplying mesentery was separated into 2.5-cm segments and rotated 90° to form an ileal plate, which was then re-tubulized along a 16-Fr. Foley tube. The newly form ileal tube was placed intraperitoneally, while the proximal end was poked through a mesocolon to anastomize the renal pelvis. The renal pelvis was then incised with a 1.5-cm opening, and the proximal ileal tube and the renal pelvis opening were anastomosed with 4–0 non-reabsorbable sutures. Anastomosis between the distal end of the ileal tube and the urinary bladder was then performed. Two JJ tubes were inserted into ileal tube during the procedure as stenting. Anti-reflux procedure using the Lich–Gregoir technique was implemented. One Jackson-Pratt drainage was placed at pelvis cavity in all of our patients. This drainage served as detection of both urinary and intestinal leakage.

### Postoperative care

We generally administered a diet appropriate for post-bowel resection, from water to a soft diet after flatus. The drainage tubes were removed if acceptable. The daily urine output was recorded, and urine leakage was monitored during the hospital stay. The Foley tube was kept for more than 1 week and removed after cystography. The Jackson-Pratt drainage was removed after the patients resume normal intake and no intestinal leakage noted.

### Follow-up

The JJ tube and pre-existing nephrostomy tube were removed at the outpatient clinic. We generally left the JJ stents in place for more than 6 weeks, and for those who had received radiotherapy, prolonged stenting for more than 8 weeks was employed. After removal of the JJ stent, we evaluated the patency of the ileal tube with antegrade pyelography and then clamp and remove the Percutaneous nephrostomy(PCN). This examination served to verify the patency of ileal tube without JJ tube. PCN was then removed after confirmation of ileal tube patency.Split renal function was monitored for at least 6 months after the surgery to assess the preservation of renal function of the affected side.

### Statistical analysis

The data obtained in the study were analyzed using descriptive statistics. The Wilcoxon signed rank test was used to determine significant statistical differences using IBM Corp. Released 2017. IBM SPSS Statistics for Windows, Version 25.0. Armonk, NY: IBM Corp..

## Results

Seven patients (four men, three women) underwent Yang–Monti ileal ureter reconstruction at our hospital. The demographic clinical parameters are listed in Table 1. One patient underwent single-session bilateral ureter repair. The most common etiology of ureter injury identified was radiation therapy-related fibrosis. The median interval between ureter insult and operation was 8 months, and the median follow-up was 36.7 months. The average operation time was 11.4 h, and the average blood loss was 273 ml. As for postoperative complications, patient 1 developed a urinary tract infection after removal of the JJ tube and was later diagnosed with hydronephrosis. A JJ tube was then re-inserted and subsequently removed with no ensuing infections reported. Patient 1 was found with UPJ stenosis and underwent re-anastomosis surgery after 11 months. Patient 3 had a urinary tract infection that needed one-time admission.

**Table 1:** Baseline and perioperative characteristics of patients who underwent Yang–Monti ileal ureter reconstruction

Patient	Age (yr)	Gender	Laterality	Indication for op	Time from ureter stricture to surgery (m)	OP time (h)	Blood loss (ml)	Hospital stays (day)	No. of bowel segments
1	47	M	Right	Idiopathic ureter stricture	60	10	200	9	5
2	45	F	Right	Post R/T fibrosis	5	9.5	250	16	5
3	63	F	Bilateral	Bil. psoas muscle abscess	36	13	350	18	5
4	40	M	Right	Degloving injury s/p TUU (Rt to Lt)	8	12	100	9	5
5	60	M	Left	Degloving injury	1	10	100	16	5
6	61	F	Left	Post R/T fibrosis	168	15	710	19	6
7	65	M	Left	Post R/T fibrosis	3	10.5	200	21	6

*Op* operation, *Bil.* bilateral, *R*/*T* radiation therapy, *TUU* transureteroureterostomy, *M* male, *F* female

There was no urinary or enteric anastamotic leakage noted in all seven patients.

None of the patients developed metabolic acidosis or electrolyte imbalance. Two patients suffered from ileus during the perioperative period and at 1 year postoperatively, both of which were managed successfully with conservative treatment. Notably, these two patients had radiation therapy-related ureter fibrosis, which is possibly associated with ileus.

**Fig. 1:**
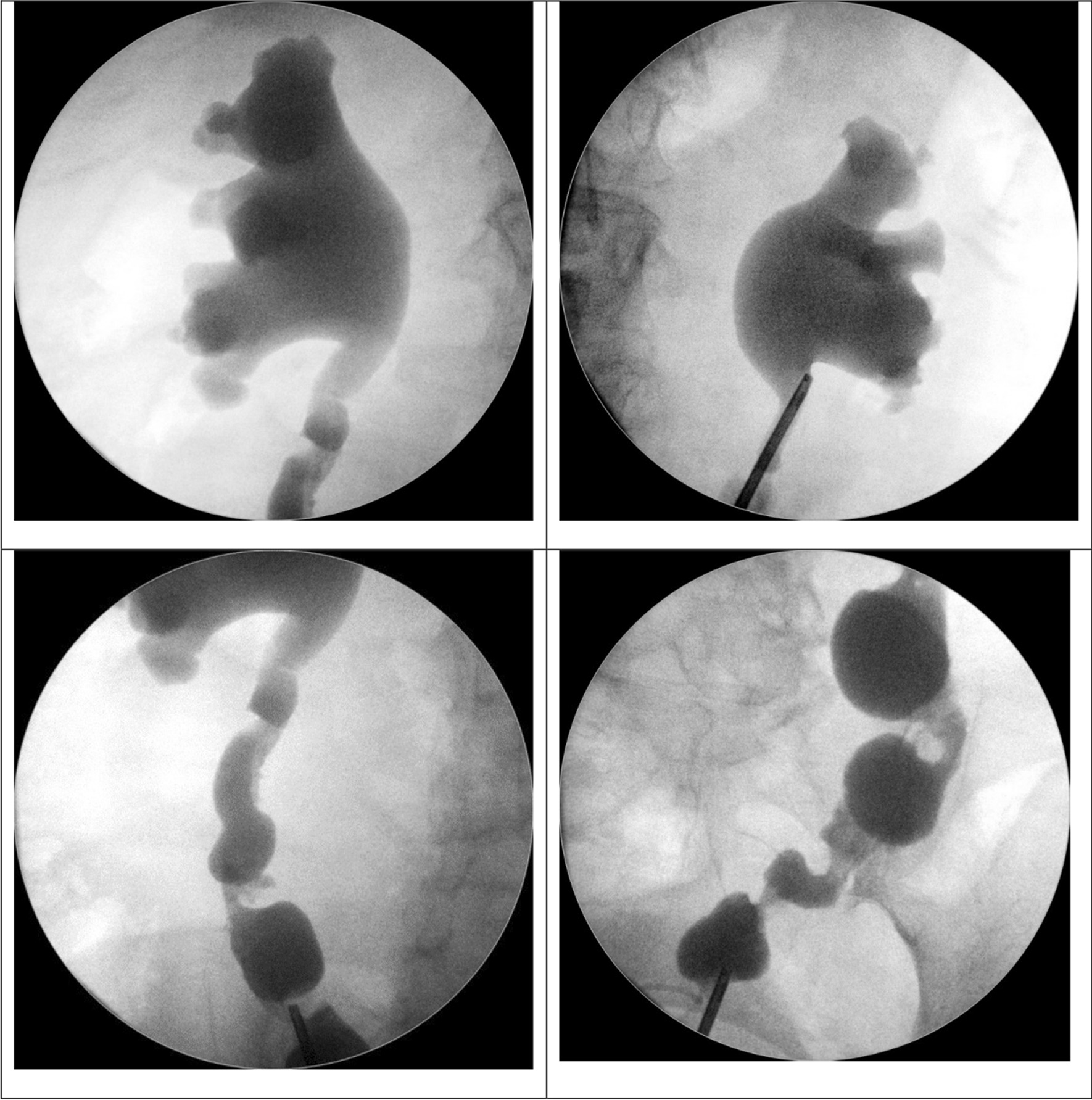
Representative retrograde pyelography (RP) images after Yang–Monti whole ureter reconstruction in patient 3. The images were taken under intraoperative C-arm at 19 months postoperatively. (Right upper and lower images) Right-side RP; (Left upper and left lower) left-side RP. Both sides showed no neo-ureter obstruction

**Fig. 2:**
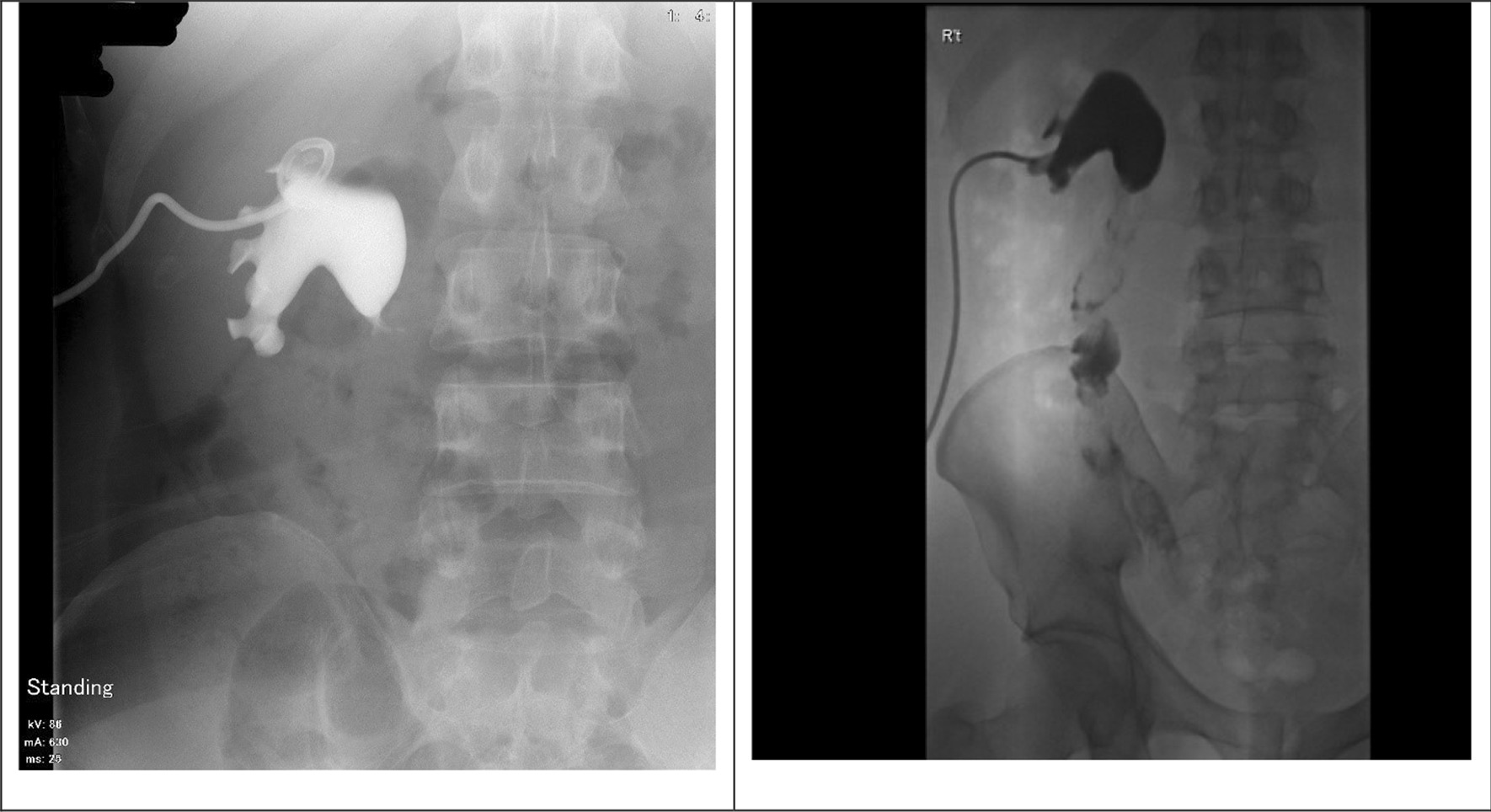
Representative antegrade pyelography before and after reconstruction. The images were the right antegrade pyelography of patient 2 taken before the operation(left) and 3 months(right) post-operatively

Due to various etiologies of ureteral obstruction and previous radiation history, a JJ tu e was kept in place for more than 8 weeks and was removed subsequently via cystoscopy. Retrograde pyelography was optional depending on the clinical condition (Fig. 1). Antegrade pyelography was obtained for each renal unit as shown in Fig. 2, and the PCNs were removed after the patency of the Yang–Monti tube was verified by antegrade pyelography. No patients underwent long-term PCN or JJ tube indwelling.

**Fig. 3:**
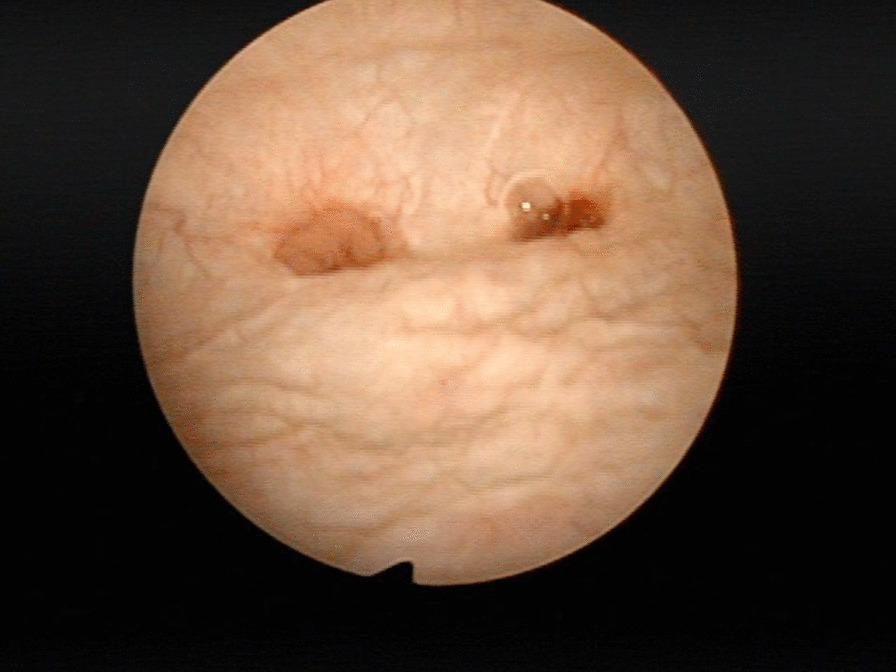
Cystoscopic images of bilateral neo-ureter orificeof patient 3 at 19 months postoperatively

**Fig. 4:**
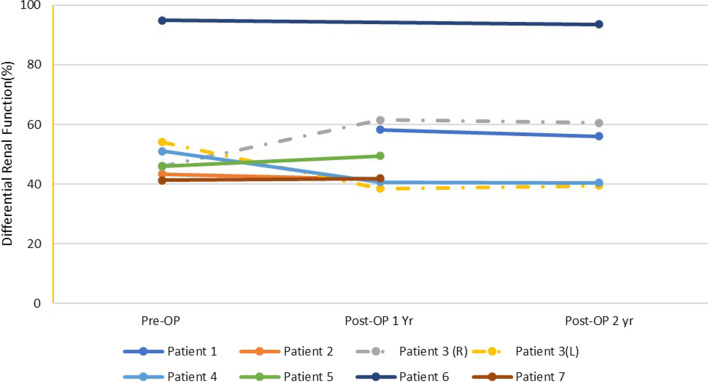
The long-term split renal function of the reconstructed side. Patient 1 had missing preoperative values. Patient 3 underwent bilateral Yang–Monti reconstruction; thus, the differential renal functions of both sides were listed separately. Pre-OP: preoperative; Post-OP 1 Yr: 1 year postoperatively; Post-OP 2 Yr: 2 years postoperatively; R: right side; L: left side

No recurrent infections, renal colic, and other symptoms/signs suggestive of ureteral obstruction were observed. Split renal function was obtained at 6 months and/or more than 12 months postoperatively, and preserved renal function was confirmed by stable renal function (Table 2, Figs. 3, 4). The p value of Wilcoxon signed rank test of split renal function was 0.83 and 0.58 at 6 and 12 months respectively compared to the baseline values.

**Table 2:** Pre- and postoperative renal function outcomes of patients with unilateral reconstruction

Patient	Serum creatinine (mg/dl)			eGFR (ml/min/1.73 m^2^)			ERPF (%)	
	Pre-OP	Post-OP 3 M	Post-OP 1Y	Pre-OP	Post-OP 3 M	Post-OP 1Y	Pre-OP	Post-OP 1Y
1	0.84	1.04	1.14	104.1	81.4	72.9		58.2
2	0.65	0.75	0.77	112.7	88.8	85.8	43.44	41.6
3	1.06	1.05	1.45	56.2	54.3	36.0	(Left) 56.2	38.5
							(Right) 43.8	61.5
4	1.13	1.1	1.01	76.4	74.0	86.5	51.1	40.7
5	1.34	1.25	1.31	57.8	62.6	59.1	46	49.5
6	1.69	1.72	1.93	31	31.93	26.22	4.96	
7	0.71	0.82	0.79	92.2	74.9	98	41.4	41.9
Mean	1.06	1.11	1.16	75.77	65.66	71.42	39.55	52.13
SD	0.34	0.32	0.39	27.12	18.91	23.59	15.96	7.16

*eGFR* estimated glomerular filtration rate, *ERPF* effective renal plasma flow, *SD* standard deviation, *Pre-OP* preoperative, *Post-OP 3 M*/*1Y* postoperative 3 months/1 year

## Discussion

We successfully treated seven consecutive patients (eight renal units) for total ureteral obstruction (from UPJ to UVJ) utilizing the Yang–Monti principle on ileal tube reconstruction. Furthermore, we have followed up each patient for more than 1 year with split renal function, which were found stable with no signs of worsening in each renal unit.

Some case series emphasized the clinical outcomes of total ureter reconstruction irrespective of surgical technique. The most commonly reported technique was ileal ureter [5–11]. One patient who underwent laparoscopic ileal ureter interposition had enteric anastomotic leakage and underwent subsequent nephrectomy [8]. Ou et al. [5] performed ileal ureter replacement in eight patients with ureter urothelial carcinoma, with two patients subsequently showing worsened renal function. Bhaskarapprakash et al. [9] performed the Yang–Monti method 3 weeks after two-point avulsion ureteral injury and reported no renal unit loss. However, the study lacked detailed examination results, such as split renal function or serum creatinine.

Deyl et al. [12] reported using cecal appendix as ureter replacement in one pediatric patient and reported no early complications to the date of publication. Li et al. [10] reported a novel surgical technique employing a vesical muscular flap in six patients with long or full-length (> 20 cm) ureteral defects. The authors harvested the muscular layer of the urinary bladder and configurated the tissue into a spiral tubal structure. The authors reported no deterioration of renal function in 2–4 years of postoperative follow-up.

Whole ureteral replacement may be achieved via different approaches or techniques, but each has its own limitations. For example, theoretically, a higher possibility of metabolic and infectious complications is expected with conventional ileal ureter, and a reasonable urinary bladder capacity is a prerequisite of the vesical muscular flap technique. The concept of a reconfigured small bowel for ureter reconstruction has been practiced since 1996 [13]. The ileum was the ideal bowel segment for reconstruction due to its fair mobility, small diameter, and consistent blood supply. Compared with conventional ileal ureter, Yang–Monti ileal ureters provide desirable gauge with shorter lengths of bowel harvested, which may result in a lower incidence of ileal tube dilatation, metabolic acidosis, excessive mucous production, and recurrent urinary tract infection. The technique is often employed in complicated or long ureter strictures.

To our knowledge, this is the largest series with the longest follow-up period in addressing whole ureter reconstruction using the Yang–Monti technique. No electrolyte imbalance, ileal tube obstruction, mucus obstruction, or recurrent urinary tract infection was observed. However, two patients suffered from ileus. Patient 6 had ileus 1 year after the reconstruction, whereas patient 7 had a similar episode within 90 days postoperatively. Both patients were managed by conservative treatment and were discharged after oral diet resumption. Of note, these two patients had both received radiation prior and related to ureteral obstruction, which was later managed by Yang–Monti ileal tube reconstruction.

The average operation time was 11.4 h, which is comparable to the operative time for bilateral reconstruction (13 h). The relatively long operation time in our case series was possibly related to the etiology of our cases. Given the majority of our patient cohort developed injuries from radiation therapy, moderate intestine adhesion was common during the operation, which indicates a considerable time interval required for enterolysis before the reconstruction.

There were three patients diagnosed with malignancy, and they all receive radiotherapy. Patient 2 and patient 6 received adjuvant radiotherapy for cervical cancer, and patient 7 received adjuvant radiotherapy after retropubic radical prostatectomy for locally advanced prostate cancer. The target volume in these three patients lay within pelvic cavity, and small intestines were more or less involved. This also translated into our relatively long operating time, for much of the time was spent on enterolysis. Ischemic endarteritis secondary to radiation may present as long as 30 years after the exposure, and there was no reliable tool for prediction of occurrence. Currently in our practice, we did not set an optimal time frame for reconstruction in these patients. Should the bowel perfusion were suboptimal for reconstruction during the abdominal exploration, we would abandon the scheduled surgical plan. Luckily, all our attempt was successful in terms of short- and intermediate-term functional result.

In our series, postoperative serum creatinine and split renal function showed no deterioration compared with the preoperative baselines.

One patient developed a Clavien–Dindo grade 3 complication, which was pelvic ileal anastomotic stenosis resulting in hydronephrosis, for which two ureteroscopic laser endopyelotomies were attempted. Ultimately, the ileal tube–renal pelvis junction was removed, and reanastomosis was performed 11 months after initial reconstruction. The continuity and patency of the Yang–Monti ileal tube was successfully restored.

Anti-reflux anastomosis has been associated with postoperative strictures [14]. Thus, the Lich–Gregoir technique was employed as anti-reflux in ileal–vesical anastomosis in our later six cases. However, we did not observe this complication in our series.

Our study has some limitations. First, this is a retrospective study, and one patient did not undergo baseline split renal function assessment. However, consistent follow-up renal function assessment and consistent subjective symptoms/signs monitoring were performed. Second, some late complications may have been overlooked or underestimated owing to relatively short follow-up periods. Third, our case series only included seven patients. However, considering the rarity of the condition, the present case series still involves the largest study on Yang–Monti ileal tube reconstruction with adequate follow-up.

## Conclusion

Our study showed that the Yang–Monti procedure is a feasible and effective procedure for total ureter reconstruction. To our knowledge, this is the largest series of total ureter reconstruction employing a reconfigured ileal tube using the Yang–Monti technique.

## Data Availability

All data generated or analysed during this study are included in this published article.

## Abbreviations

eGFR: Estimated glomerular filtration rate
UPJ: Ureteropelvic junction
UVJ: Ureterovesical junction
PCN: Percutaneous nephrostomy
